# Human Wharton’s Jelly—Cellular Specificity, Stemness Potency, Animal Models, and Current Application in Human Clinical Trials

**DOI:** 10.3390/jcm9041102

**Published:** 2020-04-12

**Authors:** Katarzyna Stefańska, Katarzyna Ożegowska, Greg Hutchings, Małgorzata Popis, Lisa Moncrieff, Claudia Dompe, Krzysztof Janowicz, Wojciech Pieńkowski, Paweł Gutaj, Jamil A. Shibli, Walterson Mathias Prado, Hanna Piotrowska-Kempisty, Paul Mozdziak, Małgorzata Bruska, Maciej Zabel, Bartosz Kempisty, Michał Nowicki

**Affiliations:** 1 Department of Histology and Embryology, Poznan University of Medical Sciences, 60-781 Poznan, Poland; k.stefanska94@o2.pl (K.S.); l.moncrieff.16@abdn.ac.uk (L.M.); claudia.dompe.16@abdn.ac.uk (C.D.); mnowicki@ump.edu.pl (M.N.); 2 Department of Infertility and Reproductive Endocrinology, Poznan University of Medical Sciences, 60-535 Poznan, Poland; katarzyna.ozegowska@ump.edu.pl; 3 Department of Anatomy, Poznan University of Medical Sciences, 60-781 Poznan, Poland; g.hutchings.16@abdn.ac.uk (G.H.); malgorzatapopis111@gmail.com (M.P.); krzysztof.janowicz.16@abdn.ac.uk (K.J.); mbruska@ump.edu.pl (M.B.); 4 School of Medicine, Medical Sciences and Nutrition, University of Aberdeen, Aberdeen AB25 2ZD, UK; 5 Division of Perinatology and Women’s Diseases, Poznan University of Medical Sciences, 60-535 Poznan, Poland; wpienkowski@ump.edu.pl; 6 Division of Reproduction, Department of Obstetrics, Gynecology, and Gynecologic Oncology, Poznań University of Medical Sciences, 33 Polna St, 60-535 Poznań, Poland; pawelgutaj@ump.edu.pl; 7 Department of Periodontology, Dental Research Division, Guarulhos University, Guarulhos, 07023-070 São Paulo, Brazil; jashibli@yahoo.com (J.A.S.); mathiasprado@uol.com.br (W.M.P.); 8 Department of Toxicology, Poznan University of Medical Sciences, 61-631 Poznan, Poland; hpiotrow@ump.edu.pl; 9 Physiology Graduate Program, North Carolina State University, Raleigh, NC 27695, USA; pemozdzi@ncsu.edu; 10 Division of Histology and Embryology, Department of Human Morphology and Embryology, Wroclaw Medical University, 50-368 Wroclaw, Poland; mazab@ump.edu.pl; 11 Division of Anatomy and Histology, University of Zielona Góra, 65-046 Zielona Góra, Poland; 12 Department of Obstetrics and Gynecology, University Hospital and Masaryk University, 602 00 Brno, Czech Republic; 13 Department of Veterinary Surgery, Institute of Veterinary Medicine, Nicolaus Copernicus University in Torun, 87-100 Torun, Poland

**Keywords:** stem cells, Wharton’s jelly, umbilical cord

## Abstract

Stem cell therapies offer a great promise for regenerative and reconstructive medicine, due to their self-renewal and differentiation capacity. Although embryonic stem cells are pluripotent, their utilization involves embryo destruction and is ethically controversial. Therefore, adult tissues that have emerged as an alternative source of stem cells and perinatal tissues, such as the umbilical cord, appear to be particularly attractive. Wharton’s jelly, a gelatinous connective tissue contained in the umbilical cord, is abundant in mesenchymal stem cells (MSCs) that express CD105, CD73, CD90, Oct-4, Sox-2, and Nanog among others, and have the ability to differentiate into osteogenic, adipogenic, chondrogenic, and other lineages. Moreover, Wharton’s jelly-derived MSCs (WJ-MSCs) do not express MHC-II and exhibit immunomodulatory properties, which makes them a good alternative for allogeneic and xenogeneic transplantations in cellular therapies. Therefore, umbilical cord, especially Wharton’s jelly, is a promising source of mesenchymal stem cells.

## 1. Introduction

Stem cells are emerging as a powerful tool in regenerative and reconstructive medicine due to their ability to self-renew, as well as their broad range of differentiation capacity. In humans, two main types of stem cells can be distinguished, namely, embryonic stem cells (ESCs) and adult stem cells (ASCs). ESCs are isolated from the inner cell mass of the pre-implantation blastocyst and are considered pluripotent, as they have the ability to differentiate into cells of all three primary germ layers: ectoderm, mesoderm, and endoderm. However, their acquisition involves embryo destruction, which raises serious ethical concerns. Moreover, the possibility to utilize ESCs in a clinical environment seems to be limited, as they may form teratomas after the transplantation, and immune rejection may occur [1,2].

ASCs comprise hematopoietic stem cells (HSCs) and mesenchymal stem cells (MSCs), the latter being isolated from various tissue sources, such as bone marrow [3], adipose tissue [4], dental pulp [5], placenta [6], and many others. In contrast, stem cell properties have been discovered in cells that are normally not considered stem cells, such as ovarian granulosa cells [7,8] or buccal pouch mucosal cells [9]. Recently, the perinatal tissues, such as the umbilical cord, have become of interest in regards to cellular therapies; because they are typically discarded after birth, their utilization as an MSC source is not ethically controversial and their collection does not involve a painful procedure. Moreover, it is suggested that MSCs from such neonatal tissues may have higher stemness potential than other MSCs [10]. Apart from this, contrary to bone marrow-derived MSCs (BM-MSCs), they do not exhibit contact-inhibited cell growth and their proliferation rate is higher than for BM-MSCs [11].

Induced pluripotent stem cells (iPSCs) offer an alternative to the stem cells naturally occurring in the organism, as they are engineered from adult, differentiated somatic cells (for example fibroblasts), and therefore their acquisition is not ethically controversial. The induction of transcription factors, such as Oct-3/4 (Octamer-binding transcription factor 3/4), Sox2 (SRY-box transcription factor 2), Klf4 (Kruppel-like factor 4), or c-Myc (MYC proto-oncogene, bHLH transcription factor), results in gaining pluripotency features by these cells [12]. Such genetic reprogramming involves a genome-wide change of DNA methylation and histone modifications, such as changes in H3K4me2 pattern [13]. However, similar to ESCs, their clinical use may pose a threat for patients, as they are prone to form teratomas in vivo [12]. Another important consideration is the fact that such iPSCs may inherit epigenetic memory from the donor tissue, which would affect their properties [14]. This was exemplified in the study by Bar-Nur et al. [15], who generated iPSCs from human pancreatic islet beta cells, and revealed that such cells maintained an open chromatin structure at key beta-cell genes, and also exhibited a unique DNA methylation signature, compared with ESCs.

Although MSCs have been isolated from various compartments of the umbilical cord, Wharton’s jelly seems to be the best source of clinically utilizable stem cells [16]. The histological structure of the umbilical cord, Wharton’s jelly-derived MSC (WJ-MSC) stemness properties, as well as the animal studies and clinical trials with their utilization are also important considerations.

## 2. Histological Structure and Cellular Composition of Umbilical Cord

**Figure 1:**
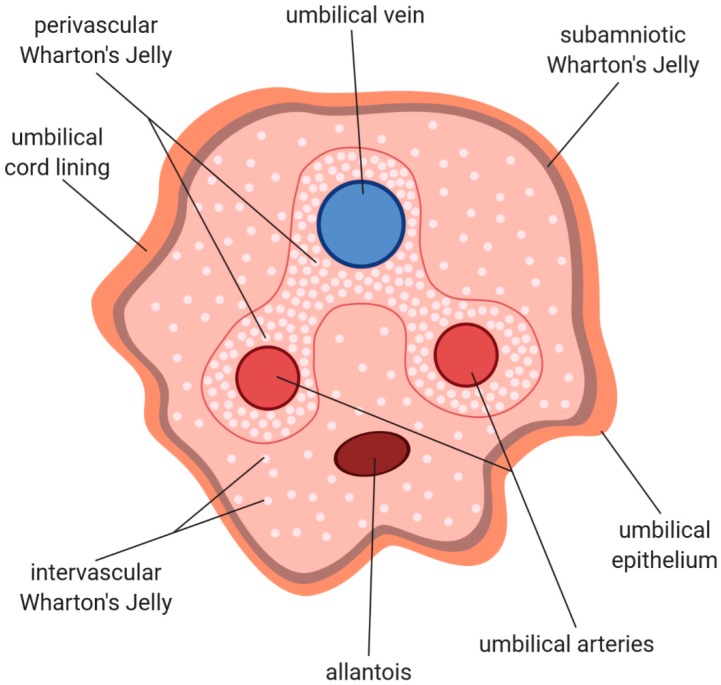
The schematic cross section of human umbilical cord covered with the umbilical cord lining, with an outer layer of umbilical epithelium, and three umbilical vessels embedded in Wharton’s jelly. Both umbilical vein and umbilical arteries are devoid of tunica adventitia. Between the latter, the residual allantois is located. Wharton’s jelly is a gelatinous connective tissue composed of extracellular matrix abundant in glycosaminoglycans (mostly hyaluronic acid), collagen fibers and myofibroblasts, and occasional mast cells. Stromal cells of Wharton’s jelly are most abundant in the proximity of the umbilical vessels in perivascular Wharton’s jelly, becoming less abundant in intervascular Wharton’s jelly, with the least amount of stromal cells in subamniotic Wharton’s jelly. The degree of stromal cells’ differentiation towards myofibroblasts is the highest near the vessels, gradually decreasing towards the umbilical epithelium.

Human umbilical cord represents the link between the mother and the fetus in the course of pregnancy, as it connects the developing embryo or fetus to the placenta. At term, it weighs about 40 g and is 3065 cm long, with an average diameter of 1.5 cm. It starts to develop at day 26 of gestation from the yolk sac and allantois, and its main function is providing the blood supply for the fetus, as well as biological waste removal [17,18,19]. Such bidirectional blood flow between the mother and the child is established by the end of the fifth week of pregnancy [20], and occurs through three umbilical vessels—two arteries and one vein (see Figure 1)—which comprise tunica intima and tunica media layers, while lacking tunica adventitia. Apart from this, the walls of the arteries are devoid of internal and external elastic lamina, whereas the internal elastic lamina is present in the thick muscularis wall of the vein. The umbilical vein is a source of mesenchymal stem cells, as the cells isolated from the vein’s endothelium and subendothelium are of fibroblastoid shape, express markers such as CD29 (Integrin beta 1), CD13, CD44, CD49e (Integrin alpha 5), CD54 (Intercellular adhesion molecule 1), CD90 (Thy-1), HLA-class 1 (Human leukocyte antigen class 1), while not expressing CD34, CD45, CD14, GLA (Glycophorin A), HLA-DR (Human leukocyte antigen—DR isotype), CD51 (Integrin alpha V), CD61 (Integrin beta 3), CD106 (Vascular cell adhesion protein 1), and CD49d (Integrin alpha 4), and are able to differentiate into adipocytes, osteocytes, and chondrocytes under appropriate stimuli [21,22].

Between two arteries, a residual allantois is located (see Figure 1), which regresses at the sixth to eighth week of gestation, resulting in the median umbilical ligament formation [20]. Moreover, near the placental end of the umbilical cord, the interarterial anastomosis, called the Hyrtl anastomosis, is present. Its role includes the alignment of blood pressure between both arteries, as well as the protection of the placenta when artery compression occurs [23].

The role of tunica adventitia, which the umbilical vessels lack, is thought to be fulfilled by Wharton’s jelly, a mucoid connective tissue described first in 1656 by Thomas Wharton. The umbilical vessels are embedded in this gelatinous tissue, and its function is of particular significance, as it prevents vessels from compression and torsion; thus, it plays a vital role in maintaining bi-directional blood transfer between the mother and the fetus, which is critical for proper fetal development [24]. Because Wharton’s jelly is classified as a connective tissue, it is abundant in extracellular matrix, composed of glycosaminoglycans (mainly hyaluronic acid), as well as different types of collagen [25]. Apart from the amorphous ground substance, Wharton’s jelly contains the fibroblast-like connective tissue cells and occasional mast cells [26,27]. Contrary to other species, such as pig or horse, Wharton’s jelly in humans lacks neural tissue, as well as other blood or lymph vessels [18,28].

Wharton’s jelly is covered with the cord lining, with an outer layer of umbilical epithelium consisting of a single or multiple layers of epithelial cells that are assumed to be of amniotic origin, expressing a cytokeratin pattern similar to human epidermis, and therefore resembling the epidermal and dermal layers of skin [26,29,30]. Moreover, the umbilical cord epithelial cells share certain features with both the fetal epidermis and the amniotic epithelium, which is not surprising, as the umbilical cords connects the amnion to the fetal skin. Therefore, the morphological differences between the aforementioned epithelias are not distinctive [31,32]. On the other hand, the umbilical epithelium is thought to be capable of stratification or keratinization, which distinguishes it from the amniotic epithelium [31,32]. Similar to the cells obtained from other parts of the umbilical cord, umbilical epithelial cells exhibit properties characteristic for mesenchymal stem cells, such as the expression of Oct-4, Nanog, SSEA-4 (Stage-specific embryonic antigen 4), CD44, CD73, CD90, CD105 (Endoglin), and others, as well as the ability to differentiate into adipogenic, chondrogenic, and osteogenic lineages [33].

On the basis of the distribution of cytoskeletal proteins of stromal cells and extracellular matrix components, Nanaev et al. [25] divided the umbilical cord’s stroma into three zones, namely, the subamniotic zone, Wharton’s jelly, and the adventitia of blood vessels, even though the typical adventitia is not present in the umbilical vessels. The division of the umbilical cord into distinct zones was further expanded by Can et al. [26], who distinguished surface epithelium, subamniotic stroma, clefts, intervascular stroma (classically regarded as Wharton’s jelly), perivascular stroma, and vessels. It is, however, suggested, that the cells contained in the perivascular stroma are derived from Wharton’s jelly, as there is no obvious demarcation between these two regions [16]. Therefore, all the stromal tissue below the umbilical cord lining to the umbilical vessels, comprising subamniotic stroma, clefts, intervascular stroma, and perivascular stroma, is considered Wharton’s jelly [28], which is illustrated in Figure 1.

## 3. Wharton’s Jelly—Cellular Structure

The cells from human Wharton’s jelly were isolated for the first time by McElreavey et al. [34] using the explant method and characterized as fibroblasts, on the basis of their morphology and expression of cellular proteins. However, subsequent studies performed by Takechi et al. [35] revealed that these cells also possess the features of muscle cells, and therefore they are not typical fibroblasts. Wharton’s jelly-derived cells described by these authors were of fusiform or stellate shape; their cytoplasm was acidophilic and the nuclei was oval or elongated. Immunohistochemical staining revealed that these cells were positive for both actin and non-muscle myosin, which are proteins known for their contractile properties. Moreover, their level in Wharton’s jelly fibroblasts was similar to those observed in smooth muscle cells [35]. These cells from Wharton’s jelly were positive for two members of the intermediate filament protein family, namely, desmin, which is one of the earliest myogenic markers in the heart and somites [35,36], and vimentin, a protein characteristic for cells of mesenchymal origin, such as fibroblasts [35,37]. Because the cells present in Wharton’s jelly possess features of both fibroblasts and muscle cells, they have been named myofibroblasts, which were initially reported to participate in wound contraction [38]. However, their mitochondrial content is not as abundant as in typical smooth muscle cells [27], and it is hypothesized that they are derived from fibroblasts rather than muscle cells [35].

The issue of their differentiation from fibroblasts to smooth muscle cells was investigated by Nanaev et al. [25], who utilized immunohistochemical staining and electron microscopy to study the characteristics of these stromal cells. The major focus of this study was to determine cytoskeletal expression patterns of vimentin, desmin, α-smooth muscle actin, γ-smooth muscle actin, and smooth muscle myosin, which would reflect the degree of their differentiation. The results indicated that the most differentiated cells were located in the proximity of the umbilical vessels, whereas the least differentiated ones were present in the subamniotic zone. Moreover, the differentiation of stromal cells occurs in a timely manner during gestation, as in the first and second trimester of pregnancy, the cells were less differentiated, meaning that their cytoskeleton expression pattern was less complex [25]. These findings were later confirmed by Kobayashi et al. [39], whose microscopic study revealed that Wharton’s jelly stromal cells started to differentiate into myofibroblasts in the second trimester of gestation, as in the first trimester the cells did not express α-smooth muscle actin, which is associated with the degree of myofibroblast differentiation.

The stromal cells are not evenly distributed throughout the umbilical cord; the microscopic studies performed by Takechi et al. [35] revealed that they were the most abundant in the proximity of umbilical vessels, becoming less abundant towards the umbilical lining, which is illustrated in Figure 1. Subsequent studies by Schugar et al. confirmed these results, indicating that the perivascular region of Wharton’s jelly may even contain 45% of all stromal cells [28,40].

Wharton’s jelly myofibroblasts are abundant in rough endoplasmic reticulum with dilated cisternae and have a well-developed Golgi apparatus. Therefore, it is assumed that the myofibroblasts are involved in synthesis and secretion of extracellular matrix protein (such as collagen) [27]. The extracellular matrix of Wharton’s jelly is rich in loosely arranged collagen fibers, whereas the elastic fibers are absent [41]. The collagen fibers become more abundant and oriented circularly around the umbilical vessels. Most of the collagen fibers are composed of type I collagen. However, as demonstrated by Sobolewski et al., there is also a relatively high level of type III collagen and a lower amount of type V collagen. About 1% of total Wharton’s jelly collagen remained unidentified in the aforementioned study [42]. However, it was hypothesized that it might be type VII collagen, expressed predominantly by epithelial cells [42], which was in agreement with earlier studies reporting its expression in cultured Wharton’s jelly stromal cells [43]. Apart from the collagen fibers, there is also a microfibrillar network present in Wharton’s jelly extracellular matrix, composed of fibrillin and type VI collagen [25,44].

The collagen fibers are embedded in an amorphous ground substance composed of glycosaminoglycans (GAGs), with hyaluronic acid constituting almost 70% of total GAG content, whereas the remaining 30% comprise keratan sulphate, heparan sulphate, chondroitin-4-sulphate, chondroitin-6-sulphate, dermatan sulphate, and heparin [42]. Moreover, Nanaev et al. reported the presence of free basement membrane proteins such as type IV collagen and laminin in Wharton’s jelly extracellular matrix, as well as the “clefts”, which were described as homogenous spaces devoid of fibrillar components, surrounded by the myofibroblasts, collagen fibers, and basal lamina molecules [25].

Although the majority of cells in Wharton’s jelly comprise myofibroblasts at various stages of differentiation, there are also mast cells present in an average number of 180 cells per cubic millimeter of tissue. These cells are mostly located in the proximity of the umbilical vessels [45].

## 4. Stemness Specificity

**Figure 2:**
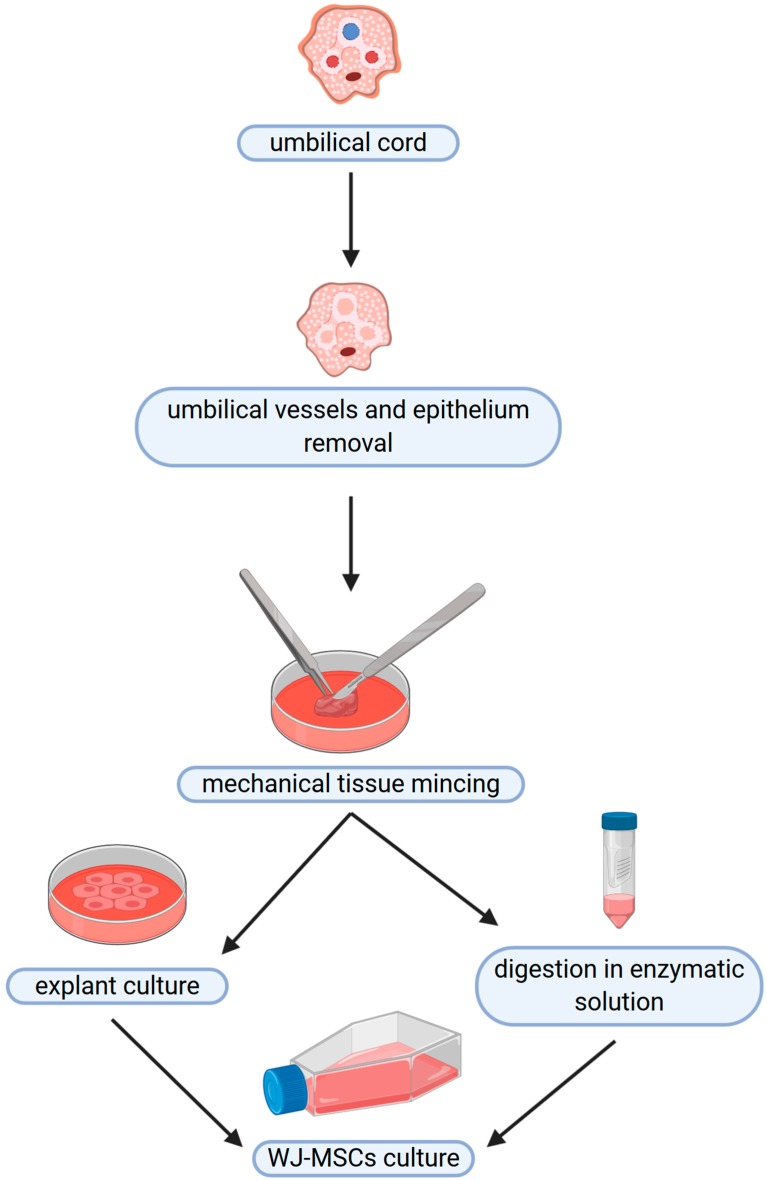
Isolation of cells from Wharton’s jelly may be performed with the use of two different methods, namely, explant method or enzymatic digestion. In both cases, the umbilical epithelium and vessels are initially removed, while the residual tissue is mechanically fragmented. The tissue pieces are placed directly on the culture vessel in the explant method, which results in the cell outgrowth on the plastic surface. In the enzymatic method, the tissue pieces are first digested in the enzymatic solution, and after that the cells released from the tissue are centrifuged, suspended in the culture medium, and seeded in the culture vessels.

The cells obtained from the umbilical cord exhibit properties characteristic for mesenchymal stromal cells (MSCs), according to the paper by the International Society for Cellular Therapy (ISCT) [46]. The minimal criteria for defining multipotent mesenchymal stromal cells include plastic adherence in standard culture conditions; expression of specific surface antigens, namely, CD105, CD73 and CD90, while not expressing CD45, CD34, CD14, CD11b (Integrin alpha M), CD79α, CD19, and HLA-II by at least 95% of the cell population; as well as the ability to differentiate into osteoblasts, adipocytes, and chondroblasts [46]. MSCs were isolated for the first time from the bone marrow by Friedenstein et al. [3] and, subsequently, from a number of adult tissues. The isolation of the cells from the umbilical cords may be performed with the use of two different methods, namely, explant method or enzymatic digestion, as illustrated in Figure 2. The explant method involves mechanical tissue mincing followed by placing the tissue at the substrate/tissue interface, which results in the cell outgrowth on the plastic surface. The enzymatic digestion method requires an additional step of fragmented tissue incubation in enzymatic solution before the transfer to the culture dishes [47].

MSCs have been isolated from various compartments of the umbilical cord, including a subendothelial layer of the umbilical vein [21,22,48,49], umbilical cord lining [33,50], and Wharton’s jelly ([51,52] among many others) with its perivascular zone as well [53,54,55]. Wharton’s jelly is the most popular source of MSCs from the umbilical cord compartments mentioned above, and was reported to be particularly rich in MSCs—the cell number in this tissue may reach up to 4,700,000 MSCs/cm of the umbilical cord; moreover, the cells exhibit a proliferative character and a short doubling time [16,18,56]. The study conducted by Subramanian et al. revealed that the cells contained in this region of the umbilical cord are the richest in stem cell properties [16].

Apart from the fact that the umbilical stromal cells fit the minimal criteria for MSCs, they also express proteins considered as markers of pluripotency and self-renewal in primitive stem cells, such as Oct-4, Sox-2, Nanog, Rex-1 (Zinc finger protein 42), SSEA-1, SSEA-4, and nucleostemin, which are also called embryonic stem cell markers [57,58,59,60]. The expression of integrin markers such as CD29 or CD51 [61]; adhesion molecules such as CD44 or CD146 [53]; and CD24 and CD108, which are assumed to confirm the presence of MSCs [16], was reported as well.

Because the stromal cells in Wharton’s jelly are of extraembryonic mesodermal origin, and their differentiation into osteoblasts, adipocytes and chondroblasts is required for WJ-derived cells to be considered mesenchymal stem cells [46], such differentiation has been conducted by many authors [16,61,62,63,64,65]. As demonstrated by Karahuseyinoglu et al. [66], Wharton’s jelly-derived MSCs (WJ-MSCs) exhibit greater differentiation potential towards chondrogenic and osteogenic lineages than bone marrow-derived MSCs. The latter, however, are more successful in adipocyte formation [66].

The potential of these cells to transform into cardiac and skeletal muscle lineages are important considerations. A successful differentiation towards cardiomyocytes using 5-azacytidine or cardiomyocyte-conditioned medium has been achieved, which was confirmed by positive expression of both cardiac troponin I and N-cadherin [61]. Conconi et al. [67] demonstrated that Wharton’s jelly-derived cells are able to differentiate into skeletal muscle cells both in vitro and in vivo and express Myf5 (Myogenic factor 5) and MyoD (Myoblast determination protein 1).

A number of studies aimed to examine the neurogenic differentiation potential of WJ-MSCs. Mitchell et al. [68] utilized a complex and multistep procedure of neurogenic induction using bFGF (Basic fibroblast growth factor), BHA (butylated hydroxyanisole), and DMSO, which resulted in morphological changes, as well as in expression of β-III tubulin, neurofilament, neuron-specific enolase, and tyrosine hydroxylase (a marker for catecholaminergic neurons). Similar results were obtained in subsequent studies. Fu et al. [69] induced neurogenic differentiation of WJ-MSCs by using neuron-conditioned medium and observed the highest expression of neuron-specific markers on the ninth day post-induction. Both aforementioned authors found that NeuN (Fox-3; neuron-specific protein) is expressed in untreated WJ-MSCs, which may indicate their intrinsic potential to differentiate along the neural program [68,69]. Liang et al. [70], on the other hand, reported WJ-MSC differentiation into cholinergic-like neurons in vitro.

Zhang et al. [62] reported using hepatic growth factor (HGF) and fibroblast growth factor-4 (FGF-4) for hepatocyte differentiation and, indeed, such induced cells expressed the hepatocyte-specific markers such as albumin, human alpha-fetoprotein, and cytokeratin 18, and were able to store glycogen and uptake LDL (low-density lipoprotein). Bharti et al. [65] also demonstrated the ability of WJ-MSCs differentiated towards hepatocytes to metabolize ammonia and produce urea. A success was also noted in obtaining pancreatic islet-like cell clusters from WJ-MSCs, which were capable of insulin release and contained human C-peptide after stepwise culturing in neuron-conditioned medium and stem cell-conditioned medium [71].

The study performed by Wu et al. [72] revealed that, after cultivation of umbilical cord-derived stem cells in the presence of vascular endothelial growth factor (VEGF) and basic fibroblast growth factor (bFGF), the MSCs differentiate into endothelial cells and express mature endothelial cell markers, such as CD31 (PECAM) or CD34. Such treated cells also gained endothelial cell function, as they were able to uptake ac-LDL. Additionally, the successful differentiation of WJ-MSCs into germ-like cells was achieved by Huang et al. [73] and confirmed with the expression of germ cell-specific markers, such as Oct4, CD117 (C-kit), CD49_f_, Stella (DDPA3) or Vasa (DDX4), whereas Hu et al. [74] reported these cells’ differentiation into bulbous cells expressing the retinal progenitor cell markers Rx (Retinal homeobox protein) and Pax6 (Paired box 6).

**Figure 3:**
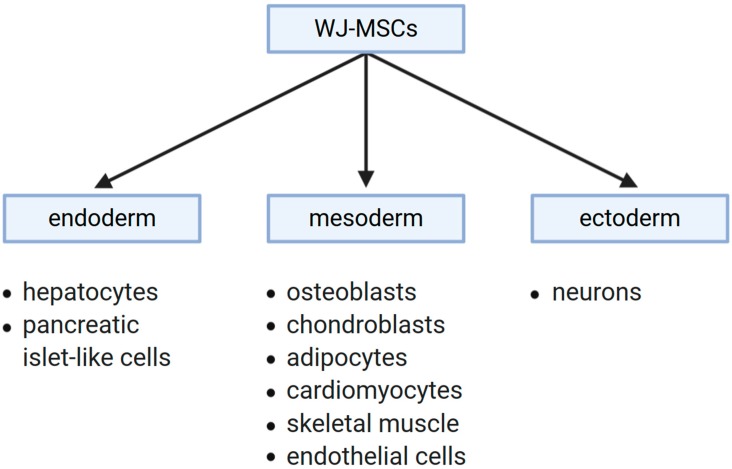
Stromal cells from Wharton’s jelly have a broad differentiation capacity and are able to transform into cells of all three primary germ layers: endoderm, ectoderm, and mesoderm. The endodermal lineages obtained from WJ-MSCs include hepatocytes or pancreatic islet-like cells. Differentiation of WJ-MSCs towards mesodermal lineages resulted in obtaining cells such as osteoblasts, chondroblasts, adipocytes, cardiomyocytes, skeletal muscle cells, or endothelial cells. Transformation of WJ-MSCs into neurons, which are of ectodermal origin, has also been achieved.

Taken together, the aforementioned findings illustrate a broad differentiation capacity of WJ-MSCs towards the cells of all three primary germ layers, as indicated in the diagram of Figure 3.

## 5. Immunomodulatory Properties of WJ-MSCs

Wharton’s jelly seems to provide a particularly attractive source of mesenchymal stem cells for regenerative and reconstructive medicine. Reports regarding HLA-ABC (also called major histocompatibility complex I—MHC-I) expression are, however, inconsistent amongst researchers; Xu et al. [63] found that 77% of WJ-MSCs expressed HLA-ABC, whereas a study by Zhou et al. [75] indicated that less than 30% of WJ-MSCs expressed this antigen. On the other hand, WJ-MSCs do not express HLA-DR (MHC-II), which makes them promising candidates for allogeneic and xenogeneic transplants [63].

Weiss et al. [52] were the first to report immunomodulatory properties of these cells in vitro, demonstrating that they suppress rat splenocyte and human peripheral blood mononuclear cell proliferation and do not stimulate proliferation of allogeneic or xenogeneic immune cells. Moreover, this study indicated that WJ-MSCs produce HLA-G6, which is an immunosuppressive form of human leukocyte antigen, inhibiting the cytolytic activity of NK cells, while not expressing immune response-related antigens involved in T lymphocyte activation, such as CD40, CD80, or CD86 [52]. The previously mentioned study by Zhou et al. [75] confirmed these findings, indicating that WJ-MSCs inhibit proliferation of both mouse splenocytes and human peripheral blood lymphocytes. Such an effect was obtained when WJ-MSCs were co-cultured with immune cells or when only WJ-MSCs culture supernatant was used. Therefore, it was hypothesized that WJ-MSCs inhibit these cells’ proliferation both via direct cell-to-cell interaction and soluble factors. Moreover, after co-culture, the secretion of TGF-β1 (Transforming growth factor β1) and IFN-γ (Interferon γ) by lymphocytes was reduced [75]. Karaöz [76], on the other hand, demonstrated upregulation of pro-apoptotic genes, such as BCL2L10 (Bcl-2-like protein 10), BAK1 (Bcl-2-like protein 7), and BIK (Bcl-2-interacting killer), after WJ-MSC and T cell co-culture, with simultaneous decrease in anti-apoptotic genes’ (BCL-2, BIRC3 (Baculoviral IAP repeat containing 3)) expression, suggesting WJ-MSCs’ apoptotic properties towards activated T cells. Deng et al. [77] reported that mesenchymal stem cells isolated from the umbilical cord secrete IL-6 (Interleukin 6), which enables them to instruct dendritic cells to acquire tolerogenic phenotypes. Other soluble factors secreted by MSCs that are involved in immunoregulation include TGF-β1, IL-10 (Interleukin 10), HGF (Hepatocyte growth factor), PGE2 (Prostaglandin E2), IDO (Indoleamine 2,3-dioxygenase), galectin-1, or HLA-G5 [52,78] Additionally, Donders et al. reported that WJ-MSCs exhibit an upregulated expression of anti-inflammatory molecules such as CD200 or PD-L1 (Programmed death ligand 1) [79].

Taking these findings into consideration, WJ-MSCs undoubtedly exhibit broad immunomodulatory properties, both via direct cell-to-cell contact and secreted soluble factors, which affect T cells’ activity. Moreover, contrary to embryonic stem cells, WJ-MSCs do not induce tumorigenesis or inflammation after transplantation, as demonstrated in the study performed by Gauthaman et al. [80]. It is, however, important to consider that immunomodulatory effects exerted by WJ-MSCs may differ between the samples, due to intrinsic variability, as demonstrated by Paladino et al. [78].

## 6. Selected Animal Models

Given the broad immunomodulatory properties of mesenchymal stem cells obtained from Wharton’s jelly, as well as their high differentiation capacity, many animal studies have been conducted in order to determine possible benefits from utilizing WJ-MSCs in treatment of diseases such as Parkinson’s disease, spinal cord injury, hindlimb or brain ischemia, diabetes, tissue fibrosis, myocardial infarction, skin regeneration, or cancer, among others, all summarized in Table 1.

Both Weiss et al. [81] and Fu et al. [82] utilized cells isolated from Wharton’s jelly in a rat model of Parkinson’s disease (PD). In the first study, the rats were injected with 6-hydroxydopamine to the medial forebrain bundle, which led to motor abnormalities similar to those seen in PD. The tested group was transplanted with approximately 1000 WJ-MSCs and received no immunosuppressive therapy. After 4 weeks, a significant decrease in apomorphine-induced rotations (which indicate motor deficits in hemiparkinsonian rats) was observed in the tested group compared to the control. A possible explanation of such results has been proposed, namely, dopaminergic neurons may have been saved from degeneration by growth and neurotrophic factors secreted by WJ-MSCs. Importantly, the cells transplanted into normal rats did not cause either immune rejection response or tumors [81]. The other previously mentioned authors, however, differentiated WJ-MSCs into dopaminergic neurons in vitro before the transplantation into the rat stratum damaged by 6-hydroxydopamine. Similar to the results obtained by Weiss et al., Fu et al. observed significant improvement in the rotational behavior in the transplanted group compared to the control [82]. Taken together, these results provide a promising alternative for Parkinson’s disease therapy and management. However, further studies have to be performed to assess possible side effects.

Because WJ-MSCs exert neuroprotective and neurotrophic effects, attempts have been made to also utilize them in spinal cord injury treatment. After the intrathecal transplantation of WJ-MSCs into rats with spinal cord injury, in several studies, the observed effects were similar, namely, an improved locomotor recovery and higher amount of spared gray matter compared to the controls [10,83,84]. Krupa et al. [83] tested two dosages of cells (0.5 M and 1.5 M), which were administered one to three times in weekly intervals. The results indicated that the effects of treatment are dose-dependent, as 1.5 M of cells provided a better outcome than 0.5 M, and this was enhanced by repeated application [83]. Chudickova et al. [84] compared the results of therapy with WJ-MSCs or their culture media (CM), as beneficial effects of MSCs are exerted mainly in a paracrine manner. Compared to the treatment with stem cells, the use of culture medium improved axonal sprouting and reduced the number of reactive astrocytes; therefore, the use of CM instead of the cells themselves seems to be an attractive alternative for spinal injury treatment [84]. Mohamadi et al. [10] focused on the molecular aspects of WJ-MSCs’ anti-inflammatory potential. They found that stem cell administration caused a decrease in expression of inflammasome complex components, illustrating an anti-inflammatory potential of WJ-MSCs [10].

The possibility of using WJ-MSCs in therapeutic angiogenesis and re-endothelialization of engineered tissue grafts has also been proposed. Wu et al. [72] demonstrated that umbilical cord-derived stem cells exhibit properties of endothelial progenitor cells, and transplanted these cells into a hindlimb ischemia mouse model. The results indicated that these MSCs, incorporated into the ischemic limb, differentiated in vivo into mature endothelial cells and contributed to neovascularization, which resulted in perfusion improvement [72]. WJ-MSCs have also been used in a rat brain ischemia model. After intracerebral transplantation, the cells migrated towards the injured tissue and differentiated into glial, neuronal, and endothelial cells, which contributed to new vessel formation, as well as increased cortical neuronal activity [85].

Another study aimed to investigate umbilical cord-derived stem cell potential in diabetes treatment in a mouse streptozotocin-induced diabetes model. The cells obtained from the umbilical cords were differentiated into islet-like cells and encapsulated in immunoisolatory biocompatible capsule for transplantation. Compared to the mice transplanted with undifferentiated cells, the tested group exhibited increased body weight, as well as the reduction in hyperglycemia. Therefore, experimentally induced diabetes was successfully reversed, and such results persisted for 3 months [86].

Another condition that was hypothesized as being of benefit from WJ-MSC cellular therapy is tissue fibrosis. Moodley et al. [87] injected these cells systemically into a bleomycin-induced mouse model of lung injury. After 2 weeks, the cells were observed as being located only in the areas of inflammation and fibrosis, which resulted in reduced collagen concentration, as well as inhibition of proinflammatory cytokine expression [87]. Tsai et al. [88] injected WJ-MSCs directly into the livers of rats with carbon tetrachloride-induced liver fibrosis, resulting in a reduction in inflammation and collagen content. This inflammatory reduction was due to the secretory activity of WJ-MSCs rather than their differentiation into hepatocytes, as they did not express either human albumin or α-fetoprotein [88]. The ability of WJ-MSCs to stimulate tissue regeneration has been studied in the SCID (severe combined immunodeficient) mouse model of skin injury [89]. The cells collected from the human umbilical cord were seeded onto decellularized amniotic membrane scaffold and then grafted onto the injured skin, which resulted in improvement of biomechanical properties of the regenerated skin, as well as in reduced scar formation and better healing abilities in general [89].

Several studies aimed to investigate whether WJ-MSC transplantation may be a viable method to treat myocardial infarction. Zhang et al. [90] demonstrated the fate of the stem cells that were injected into the sites of ischemia in acute myocardial infarction mini-swine model, where they were reported to have differentiated towards both cardiomyocytes and endothelial cells and promoted recruitment and differentiation of cardiac stem cells. Cellular apoptosis was reduced after 6 weeks, and ventricular remodeling and function was improved [90]. Nascimento et al. [91] performed a similar experiment on a mouse model of myocardial infarction, and the transplanted cells (UCX) were isolated from umbilical cords using patented proprietary technology. The results confirmed that which was observed in the previous study, namely, the reduced apoptosis in the injured tissue, increased capillary density, and general cardioprotective effects. However, it was hypothesized that these cells exert paracrine effects on cardiac tissue, as there was no differentiation towards cardiomyocytes [91].

Several studies focused on utilization of WJ-MSCs in treatment of various types of cancer. Matsuzuka et al. [92] demonstrated that the cells isolated from Wharton’s jelly were transfected with IFN-β gene, as interferon β exhibits anti-tumor properties. Such modified stem cells inhibited the growth of bronchioloalveolar carcinoma cell lines when co-cultured. A similar effect was obtained by using conditioned culture medium. Moreover, after systemic injection of modified WJ-MSCs into SCID mice xenografted with bronchioloalveolar carcinoma, the tumor growth was reduced as a result of cell apoptosis [92]. Ayuzawa et al. [93] and Ma et al. [94] tested the influence of naïve WJ-MSCs on human breast cancer cells in SCID mice, and discovered that intravenous [93] or local [94] injections of stem cells result in their localization in the proximity of the tumor, which leads to tumor growth attenuation [93,94]. Ma et al. hypothesized that this effect may be a result of PI3K (Phosphoinositide 3-kinase) and AKT (Protein kinase B) protein kinase activity suppression [94].

## 7. Clinical application of Wharton’s jelly

A number of preclinical and clinical studies utilizing Wharton’s jelly have been undertaken. Can et al. [95] extensively reviewed clinical trials utilizing UC-MSCs. Amongst 93 papers, 23 concerned neurological diseases, 19 hematological diseases, 15 immunological diseases, 10 liver diseases, 7 cardiac diseases, 6 endocrine diseases, 7 musculoskeletal diseases, 3 pulmonary diseases, 2 skin diseases, and 1 ophthalmological diseases, comprising both clinical trials and single case reports. However, the exact source of cells used in these trials was not specified, as they were referred to as “UC-MSCs”. In this review, we aimed to investigate clinical trials with the use of WJ-MSCs, and therefore the website clinicaltrials.gov was used, searching the terms “Wharton’s jelly mesenchymal stem cell” and “WJ-MSC”. Amongst the results, 6 clinical studies have been completed, 3 are currently ongoing, 10 are currently recruiting participants, 6 are not yet recruiting, and 1 is of unknown status. The conditions that are aimed at being treated with WJ-MSCs or their conditioned media include erectile disfunction associated with type 2 diabetes mellitus (NCT02945449, NCT03751735), retinitis pigmentosa and inherited retinal dystrophy (NCT04224207), osteoarthrosis (NCT02963727), osteoarthritis (NCT03866330, NCT03337243), diabetic nephropathy (NCT03288571), spinal cord injury (NCT03003364, NCT04288934), type I diabetes (NCT03973827, NCT03406585), acute graft versus host disease (NCT03158896), cardiovascular diseases (NCT01291329, NCT02368587, NCT04011059, NCT03404063, NCT03798353, NCT03418233, NCT03533153), amyotrophic lateral sclerosis (NCT02881476), inflammatory bowel disease (NCT03299413), critical limb ischemia (NCT03423732), chronic ulcer wounds (NCT04134676), decompensated liver cirrhosis (NCT03529136), pre-eclampsia diagnosis (NCT03562715), and COVID-19 (NCT04313322). However, to date, the articles have been published only on studies NCT01291329 and NCT02881476 among the aforementioned clinical trials.

The emphasis of Wharton’s jelly concerns the treatment of cardiovascular diseases. In the study conducted by Gao et al. (NCT01291329) [96], 116 patients with ST-elevation acute myocardial infarction received intracoronary injection of WJ-MSCs or placebo to evaluate safety and efficacy of such a treatment. In the tested group, a reduction in myocardial infarct size and improved heart function was observed when compared to the control, and left ventricular adverse remodeling was prevented. Moreover, no adverse effects such as teratoma formation or immune rejection after cell transplant were reported [96]. In the case of previously mentioned amyotrophic lateral sclerosis (ALS) (NCT02881476) [97], a group of 43 patients received WJ-MSCs once, twice, or three times, within a 2 month interval. The cells were suspended in autologous cerebrospinal fluid and administered intrathecally at the cervical, thoracic, or lumbar region. The aim of this study was to examine whether such a therapy would be safe and well tolerated by patients with ALS; no serious adverse events, apart from a headache in one case, were reported. Therefore, the researchers concluded that intrathecal injections of WJ-MSCs are safe for patients with ALS, and other outcomes of this treatment are being currently analyzed [97].

In addition to the search for clinical trials on the clinicaltrials.gov website, similar PubMed searches were performed as well. As a result, we obtained a list of more published clinical trials. The results of both searches are summarized in Table 2. Recent studies concerned the treatment of immunologic-related disorders with WJ-MSC transplantation. In a study by Zhang et al. (NCT01213186) [98], stem cells were administered to HIV-1-infected immune non-responder patients three times in monthly intervals. The observed results included an increase in the number of circulating and central memory CD4 T cells and the production of IFN-γ and IL-2, which caused general improvement in immune reconstitution. Similar to the previous studies, no adverse effects have been observed, which indicate that WJ-MSC transfusions were well tolerated in immune-deficient patients [98].

Another four studies concerned autoimmune diseases, however, the stem cells used in three of these studies were obtained from the whole umbilical cord to treat the active and refractory systemic lupus erythematosus (SLE) (NCT01741857) [99], neuromyelitis optica (NCT01364246) [100], or active rheumatoid arthritis (RA) (NCT01547091) [101]. Wang et al. [99] administered MSCs from the umbilical cord intravenously, in a weekly interval, to 40 patients with lupus. After a 12 month follow-up, the survival rate was 92.5% and the condition of the remaining patients significantly improved, according to SLEDAI (Systemic Lupus Erythematosus Disease Activity Index) and BILAG (British Isles Lupus Assessment Group) scores [99]. Such a result was explained by the secretion of indoleamine 2,3-dioxygenase by MSCs from the umbilical cord, which led to T cell proliferation suppression [102]. In case of rheumatoid arthritis, a group of 136 patients received a treatment with MSCs from the umbilical cord injected intravenously, alongside anti-rheumatic drugs. The therapy resulted in decreased levels of tumor necrosis factor-alpha and IL-6, and an increased percentage of regulatory T cells in peripheral blood, compared to the control, which led to significant remission of the disease [101]. However, it has been suggested that both treatments should be repeated after 6 months, as the clinical benefits declined after that time period [99,101]. MSCs from the umbilical cord were also used to treat neuromyelitis optica in five patients, who received intravenous and intrathecal injections of stem cells four times in weekly intervals. During an 18 month follow up, four patients exhibited an improvement in signs and symptoms, as well as reduced relapse frequencies, which was hypothesized as being the result of B cell inhibition and simultaneous increase of T cell levels [100]. In the fourth study, on the other hand, the WJ-MSCs were used to treat type 1 diabetes (T1D). The group of 15 newly onset T1D patients were administered with WJ-MSCs intravenously twice in a monthly interval, apart from a standard insulin therapy. As a result, the mean value of glycated hemoglobin significantly decreased compared to the control, whereas the mean C-peptide levels were significantly increased, and therefore it was hypothesized that such a therapy could restore the β cell function in a longer period of time [103]. Importantly, no serious side effects or adverse events were reported.

In addition to type 1 diabetes, WJ-MSC transplantation has also been performed in order to manage type 2 diabetes (T2D). A total of 23 patients with T2D received WJ-MSCs twice, intravenously for the first time and directly to the pancreas via the splenic artery for the second time. Similar to the results obtained in the case of type 1 diabetes, the C-peptide levels improved, whereas glycated hemoglobin levels decreased after treatment. The authors also reported a reduction in markers of systemic inflammation, whereas no adverse events occurred [104]. Lv et al. (NCT01343511) [105] injected cells isolated from the Wharton’s jelly twice intrathecally to nine patients with autism, alongside cord blood mononuclear cells (CBMNCs). During the 24 week follow up, there was a significant improvement in patients’ symptoms when compared to the control group, as indicated by CARS (The Childhood Autism Rating Scale), CGI (Clinical Global Impression), and ABC (Aberrant Behavior Checklist) scores. Moreover, injections of WJ-MSCs combined with CBMNCs provided a better outcome than the use of CBMNCs alone, and there were no adverse events reported [105]. WJ-MSCs also enhance hematopoiesis after cord blood transplantation, as indicated by Wu et al. [106]. Eight patients with the high-risk acute lymphoblastic leukemia or the high-risk acute myeloid leukemia were co-transplanted with WJ-MSCs and cord blood, which resulted in shorter time of neutrophil and platelet engraftment when compared to the group of patients receiving the cord blood alone. Importantly, no infusion toxicity or ectopic tissues were observed after the transplantation, and therefore it was hypothesized that WJ-MSC therapy is safe for immunocompromised patients [106].

In all of the trials mentioned above, no serious side effects or adverse events have been reported, indicating that treatment with WJ-MSCs is safe for patients. In cases where clinical improvement was reported, it was mostly due to immunomodulatory properties of WJ-MSCs. However, such claims must be treated with caution, as some of the aforementioned studies were not blinded or placebo-controlled, or, in the case of neuromyelitis optica [100], the studied group was relatively small. Even though the clinical improvements described in the aforementioned trials seem promising, it is possible that in some cases they occurred due to the placebo effect. Another issue is the fact that tracing of administered cells is problematic, and therefore their fate after the injection remains unclear. Such cells may integrate into host tissue rather than be metabolized and exerted from the body, thus safety issues may not become apparent for decades [107]. Therefore, it is essential to conduct more standardized, blinded, and placebo-controlled trials before introducing WJ-MSC treatment to patients. There is no doubt that all clinical trials should be conducted in accordance to the “Guidelines for Stem Cell Research and Clinical Translation”, published by ISCT in 2016 [108].

## 8. Conclusions

The cells obtained from Wharton’s jelly seem to provide a promising alternative as a stem cell source. Apart from meeting the minimal criteria for MSCs, they also express a number of proteins considered as embryonic stem cell markers. The broad differentiation capacity of WJ cells allows them to transform into cells from all three primary germ layers under appropriate stimuli. Apart from this, WJ-MSCs do not express MHC-II and possess unique immunomodulatory properties, which makes them excellent for allogeneic and xenogeneic transplantations. Indeed, a number of preclinical and clinical studies using WJ-MSCs have been performed to examine their potential in the management of various diseases. These stem cells were demonstrated to improve the condition of patients with cardiovascular diseases, immunologic-related disorders, or neurological diseases. The animal studies revealed that therapy with the use of WJ-MSCs may be beneficial in treatment of Parkinson’s disease, tissue fibrosis, cancer, and spinal cord injury, amongst others, whether it was due to their differentiation into cells of the damaged tissues or paracrine mechanisms. It is important to note that all of these studies were successful in improving clinical outcomes, with no evidence of teratoma formation or immune rejection. Therefore, WJ-MSCs seem to be a great promise of regenerative medicine. However, further studies are needed to consider any possible side effects of WJ therapies.

## Figures and Tables

**Table 1:** Animal studies with the use of stem cells from human umbilical cords.

Treated Condition	Animal Model	Mean Dose of Stem Cells in One Injection	Source of MSCs	Delivery Method	Author	Publication Date	Journal
Parkinson’s disease	Hemiparkinsonian Sprague-Dawley rats	1 × 10^3^	Wharton’s jelly	Intrastriatal injection	Weiss et al. [81]	2006	*Stem Cells*
	Parkinsonian Sprague-Dawley rats	1 × 10^5^	Wharton’s jelly	Intrastriatal injection	Fu et al. [82]	2006	*Stem Cells*
Spinal cord injury	Wistar rats with spinal cord injury	0.5 or 1.5 × 10^6^	Wharton’s jelly	Intrathecal infusion	Krupa et al. [83]	2018	*International Journal of Molecular Sciences*
	Wistar rats with spinal cord injury	1.5 × 10^6^	Wharton’s jelly	Intrathecal infusion	Chudickova et al. [84]	2019	*International Journal of Molecular Sciences*
	Wistar rats with spinal cord injury	3 × 10^5^	Wharton’s jelly	Intrathecal infusion	Mohamadi et al. [10]	2019	*Journal of Chemical Neuroanatomy*
Hindlimb ischemia	Athymic nude mice with hindlimb ischemia	1 × 10^6^	Umbilical cord (not specified)	Injection to the adductor muscle	Wu et al. [72]	2007	*Journal of Cellular Biochemistry*
Brain ischemia	Sprague-Dawley rats with brain ischemia	1 × 10^6^	Wharton’s jelly	Intracerebral injection	Ding et al. [85]	2007	*Neurobiology of Disease*
Diabetes	Streptozotocin-induced diabetic Balb/C mice	3 × 10^6^ of undifferentiated cells or 1 × 10^3^ islet-like clusters	Umbilical cord (not specified)	Transplantation of encapsulated cells into abdomen	Kadam et al. [86]	2010	*Islets*
Pulmonary fibrosis	Bleomycin-induced lung injury in SCID mice	1 × 10^6^	Wharton’s jelly	Intravenous injection	Moodley et al. [87]	2009	*The American Journal of Pathology*
Liver fibrosis	Sprague-Dawley rats with CCl_4_-induced liver fibrosis	5 × 10^5^	Wharton’s jelly	Injection into the right lobe of the liver	Tsai et al. [88]	2009	*Liver Transplantation*
Skin injury	SCID mice with skin injury	1 × 10^6^	Wharton’s jelly	Injection into the skin or	Sabapathy et al. [89]	2014	*PLoS One*
Myocardial infarction	Guangxi Bama miniswines with acute myocardial infarction	4 × 10^7^	Wharton’s jelly	Injection into the ischemic region of the heart	Zhang et al. [90]	2013	*Coronary Artery Disease*
	C57BL/6 mice with myocardial infarction	2 × 10^5^	Umbilical cord (not specified)	Intramyocardial injection	Nascimento et al. [91]	2014	*Stem Cell Research and Therapy*
Cancer	CB17 SCID mice with bronchioloalveolar carcinoma	3 × 10^5^	Wharton’s jelly	Intravenous injection	Matsuzuka et al. [92]	2010	*Lung Cancer*
	CB17 SCID mice transplanted with MDA 231 human breast carcinoma cells	5 × 10^5^	Wharton’s jelly	Intravenous injection	Ayuzawa et al. [93]	2009	*Cancer Letters*
	CB17 SCID mice transplanted with MDA 231 human breast carcinoma cells	0.5 × 10^6^ or1 × 10^6^ or 3 × 10^6^	Wharton’s jelly	Subcutaneous injection at the tumor site	Ma et al. [94]	2012	*Breast Cancer Research and Treatment*

**Table 2:** Clinical trials with the use of stem cells from human umbilical cords.

Treated Condition	Number of Study Participants	Type of Study	Source of Stem Cells/Number of Donors	Mean Dose of Stem Cells in One Injection	Delivery Method	Author	Publication Date	Journal
Acute myocardial infarction	116	Randomized double-blind controlled trial	Wharton’s jelly/pooled from 21 donors	6 × 10^6^	Intracoronary infusion	Gao et al. [96]	2015	*BMC Medicine*
Amyotrophic lateral sclerosis	43	Not specified	Wharton’s jelly/pooled from three donors	0.42 × 10^6^/kg of body weight	Intrathecal injection	Barczewska et al. [97]	2019	*Neural Regeneration Research*
HIV-1-infected immune nonresponders	13	Prospective open-labeled controlled trial	Wharton’s jelly/not specified	0.5 × 10^6^/kg of body weight	Intravenous infusion	Zhang et al. [98]	2013	*AIDS*
Active and refractory systemic lupus erythematosus	40	Multicenter trial	Umbilical cord (not specified)/not specified	1 × 10^6^/kg of body weight	Intravenous infusion	Wang et al. [99,102]	2014; 2014	*Arthritis Research and Therapy;* *Arthritis and Rheumatology*
Neuromyelitis optica	5	Not specified	Umbilical cord (not specified)/not specified	4 × 10^7^ or 2 × 10^7^	Intravenous and intrathecal infusion	Lu et al. [100]	2012	*Current Neurovascular Research*
Rheumatoid arthritis	172	Single center trial	Umbilical cord (not specified)/not specified	4 × 10^7^	Intravenous infusion	Wang et al. [101]	2013	*Stem Cells and Development*
Type 1 diabetes mellitus	29	Randomized double-blind controlled trial	Wharton’s jelly/one donor	2.6 ± 1.2 × 10^7^	Intravenous infusion	Hu et al. [103]	2013	*Endocrine Journal*
Type 2 diabetes mellitus	22	Single center prospective trial	Wharton’s jelly/not specified	1 × 10^6^/kg of body weight	Intravenous infusion and intra-pancreatic endovascular injection	Liu et al. [104]	2014	*Stem Cell Research and Therapy*
Autism	37	Non-randomized open-label, single center trial	Wharton’s jelly/not specified	1 × 10^6^/kg of body weight	Intravenous and intrathecal infusion	Lv et al. [105]	2013	*Journal of Translational Medicine*
High risk leukemia	20	Randomized trial	Wharton’s jelly/not specified	1 × 10^6^/kg of body weight	Intravenous infusion	Wu et al. [106]	2013	*Cell Transplantation*
